# Supplementary material for: Magnitude-sensitive reaction times reveal non-linear time costs in multi-alternative decision-making
Source: PLoS Comput Biol. 2022 Oct 3;18(10):e1010523. doi: 10.1371/journal.pcbi.1010523 (PMC9560628; doi:10.1371/journal.pcbi.1010523)
Supplement: S1 Text — (PDF) [file pcbi.1010523.s001.pdf]

Supplementary Information for

Magnitude-sensitive reaction times reveal non-linear time costs in multi-alternative decision-making

S1 Text - TASK INSTRUCTIONS

Throughout the task, participants read the following text:

Screen 1

In each trial you will be presented with three flickering grey patches. Before and during each trial please focus on the central cross. For each trial you have to decide which patch is the brightest by pressing left, up or down on the keyboard. Your score will depend on choosing the brightest patches. Try to be as fast and accurate as possible. After giving your response a new trial will start. Press spacebar to continue.

Screen 2

Please place the index finger of your right hand on the left arrow key on the keyboard, your middle finger on the up arrow, and your ring finger on the right arrow. Press spacebar to start 6 training trials. By continuing you are confirming that you understand the nature of the experiment and consent to participate. You may leave the experiment at any time by closing your browser.

Screen 3

Training trials are finished. The experiment consists of 100 trials. It will take about 5 minutes to complete. For those 5 minutes please concentrate on the task; focus on the cross throughout. When you are ready press spacebar to start the experiment.

Screen 4

The experiment is finished! Thank you for contributing to our research. Press spacebar to quit.
