# Supplementary material for: Magnitude-sensitive reaction times reveal non-linear time costs in multi-alternative decision-making
Source: PLoS Comput Biol. 2022 Oct 3;18(10):e1010523. doi: 10.1371/journal.pcbi.1010523 (PMC9560628; doi:10.1371/journal.pcbi.1010523)
Supplement: S1 Table — Participant ID was included as a random factor. The regression was performed using R (RStudio Version 1.2.1335; function ‘lmer’, package ‘lme4’). Given the typical skewness of reaction times, the dependent variable was transformed (i.e. normalized) using the ‘bestNormalize’ function in R. As the brightness of equal alternatives increased, reaction times significantly decreased. (PDF) [file pcbi.1010523.s002.pdf]

Supplementary Information for

Magnitude-sensitive reaction times reveal non-linear time costs in multi-alternative decision-making

	Reaction time		
Predictors	*Estimates*	*CI*	*p*
(Intercept)	0.87	0.73 – 1.02	**<0.001**
Brightness	-1.95	-2.14 – -1.75	**<0.001**
**Random Effects**			
σ^2	0.58		
τ_{00} ID Particiapnt	0.38		
ICC	0.39		
N ID Participant	117		
Observations	4644		
Marginal R^2 / Conditional R^2	0.047 / 0.422		

S1 Table Mixed-effect regression for reaction times as a function of the brightness of the equal alternatives in the human study. Participant ID was included as a random factor. The regression was performed using R (RStudio Version 1.2.1335; function *lmer*, package lme4). Given the typical skewness of reaction times, the dependant variable was transformed (i.e., normalized) using the *bestNormalize* function in R. As the brightness of equal alternatives increased, reaction times significantly decreased.
