# Supplementary material for: Magnitude-sensitive reaction times reveal non-linear time costs in multi-alternative decision-making
Source: PLoS Comput Biol. 2022 Oct 3;18(10):e1010523. doi: 10.1371/journal.pcbi.1010523 (PMC9560628; doi:10.1371/journal.pcbi.1010523)
Supplement: S2 Table — Sclerotia identity was included as a random factor. The regression was performed using R (RStudio Version 1.2.1335; function ‘lmer’, package ‘lme4’). Given the typical skewness of reaction times, the dependent variable was transformed (i.e. normalized) using the ‘bestNormalize’ function in R. As the food quality of equal alternatives increased, reaction times significantly decreased. (PDF) [file pcbi.1010523.s003.pdf]

Supplementary Information for

Magnitude-sensitive reaction times reveal non-linear time costs in multi-alternative decision-making

	Latency to reach the food		
Predictors	*Estimates*	*CI*	*p*
(Intercept)	1.27	0.97 – 1.56	**<0.001**
Food Quality	-0.03	-0.03 – -0.02	**<0.001**
**Random Effects**			
σ^2	0.65		
τ_{00} ID Plasmodium	0.03		
ICC	0.05		
N ID Plasmodium	10		
Observations	200		
Marginal R^2 / Conditional R^2	0.322 / 0.353		

S2 Table Mixed-effect regression for reaction times as a function of food quality in the slime moulds study. Sclerotia identity was included as a random factor. The regression was performed using R (RStudio Version 1.2.1335; function *lmer*, package lme4). Given the typical skewness of reaction times, the dependant variable was transformed (i.e., normalized) using the *bestNormalize* function in R. As the food quality of equal alternatives increased, reaction times significantly decreased.
