# Supplementary material for: Magnitude-sensitive reaction times reveal non-linear time costs in multi-alternative decision-making
Source: PLoS Comput Biol. 2022 Oct 3;18(10):e1010523. doi: 10.1371/journal.pcbi.1010523 (PMC9560628; doi:10.1371/journal.pcbi.1010523)
Supplement: S1 Fig — The top panel shows the values used in Fig 5 and SF1; the bottom panel shows the values used in Fig 6. (PDF) [file pcbi.1010523.s004.pdf]

Supplementary Information for

Magnitude-sensitive reaction times reveal non-linear time costs in multi-alternative decision-making

S1 Fig – Shape of the utility functions for different values of *s* and *m* of the logistic function of Eq. (1) in the main text. The top panel shows the values used in Fig. 5 and SF1; the bottom panel shows the values used in Fig. 6.

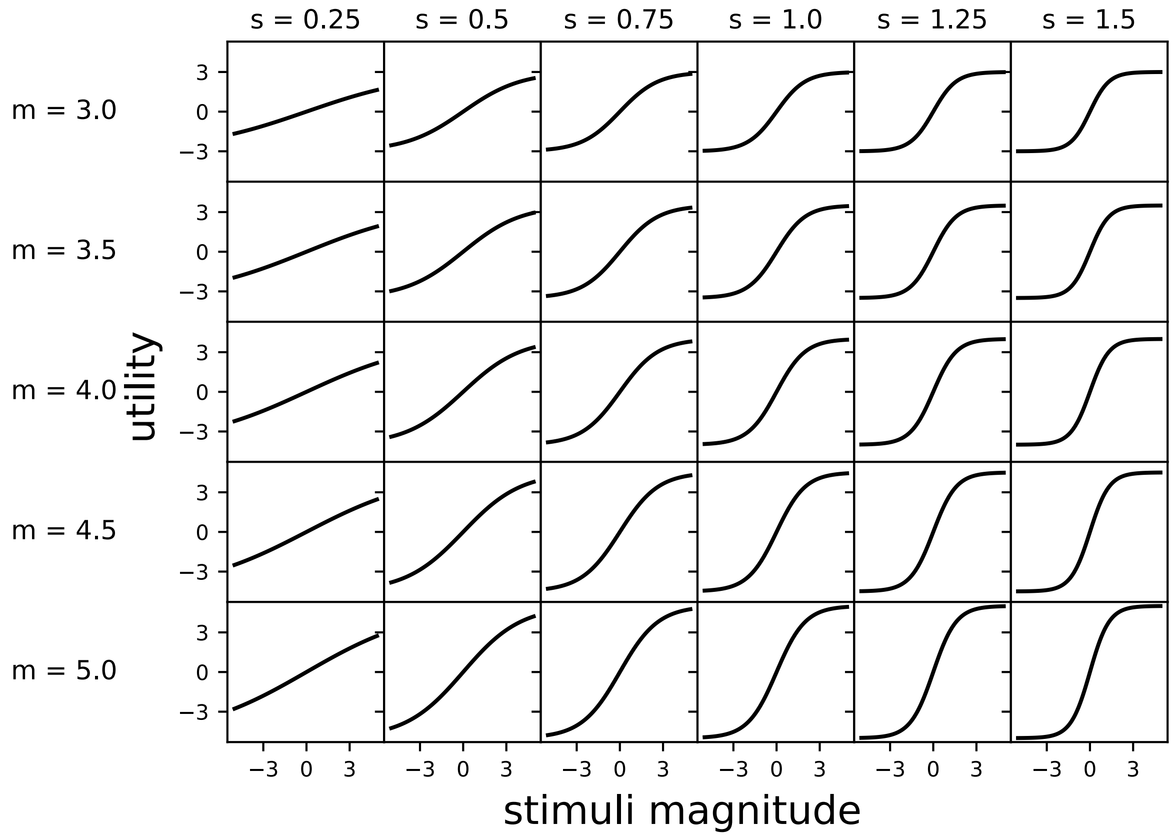

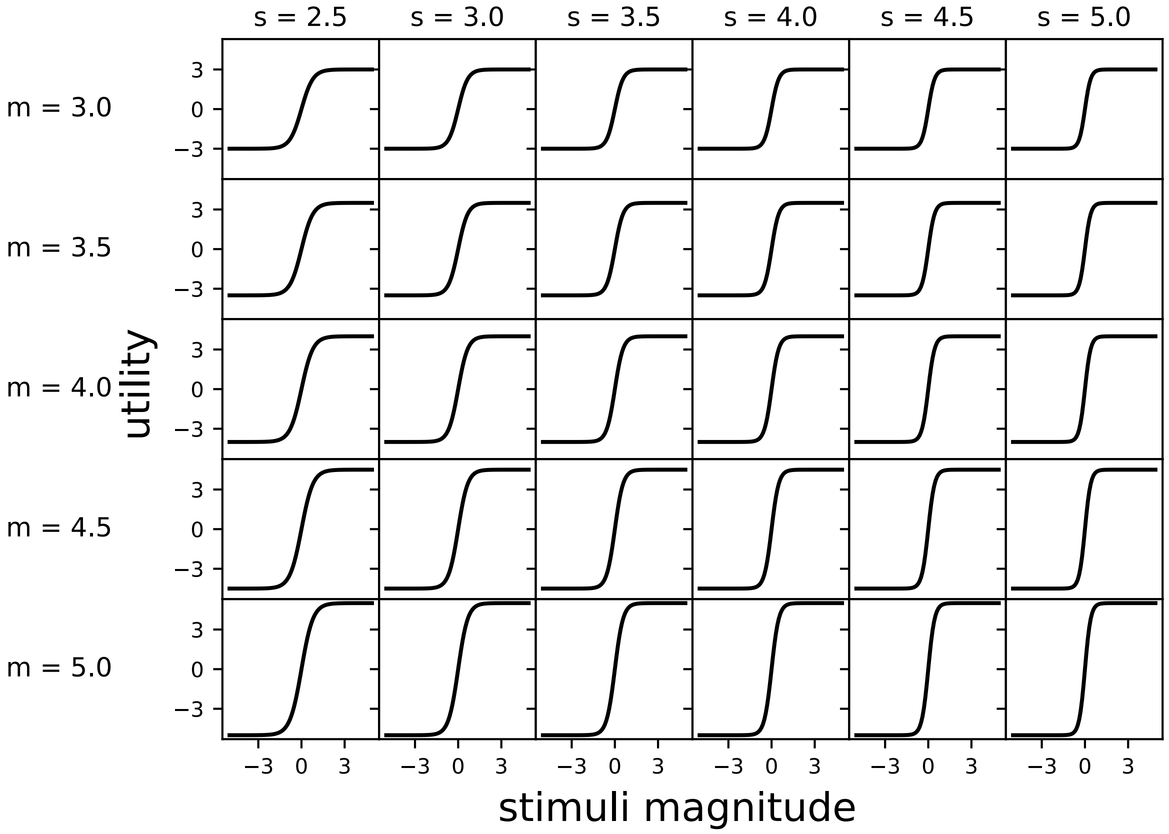
s = 2.5
s = 3.0
s = 3.5
s = 4.0
s = 4.5
s = 5.0
m = 3.0
m = 3.5
m = 4.0
m = 4.5
m = 5.0
utility
stimuli magnitude
3
0
−3
