# Supplementary material for: Magnitude-sensitive reaction times reveal non-linear time costs in multi-alternative decision-making
Source: PLoS Comput Biol. 2022 Oct 3;18(10):e1010523. doi: 10.1371/journal.pcbi.1010523 (PMC9560628; doi:10.1371/journal.pcbi.1010523)

Supplementary Information for

Magnitude-sensitive reaction times reveal non-linear time costs in multi-alternative decision-making

S2 Fig – Geometric discounting of reward leads to magnitude-sensitive simulated reaction times across a range of nonlinear subjective utility functions, with decisions postponed for low equal-value option sets. Simulation parameters were: prior mean $\bar{x}_{p,i}$ = 1.5 and variance $\sigma^2{}_{p,i}$ = 5, observation noise variance $\sigma^2{}_{a,i}$ = 2, temporal cost γ = 0.9, and simulation timestep dt = 5 × 10^{-3}. Lines are the mean reaction time for 10^4 simulations, 95% confidence intervals are shown as red shading (mostly invisible because smaller than the linewidth). Non-decision-time was implicitly zero.

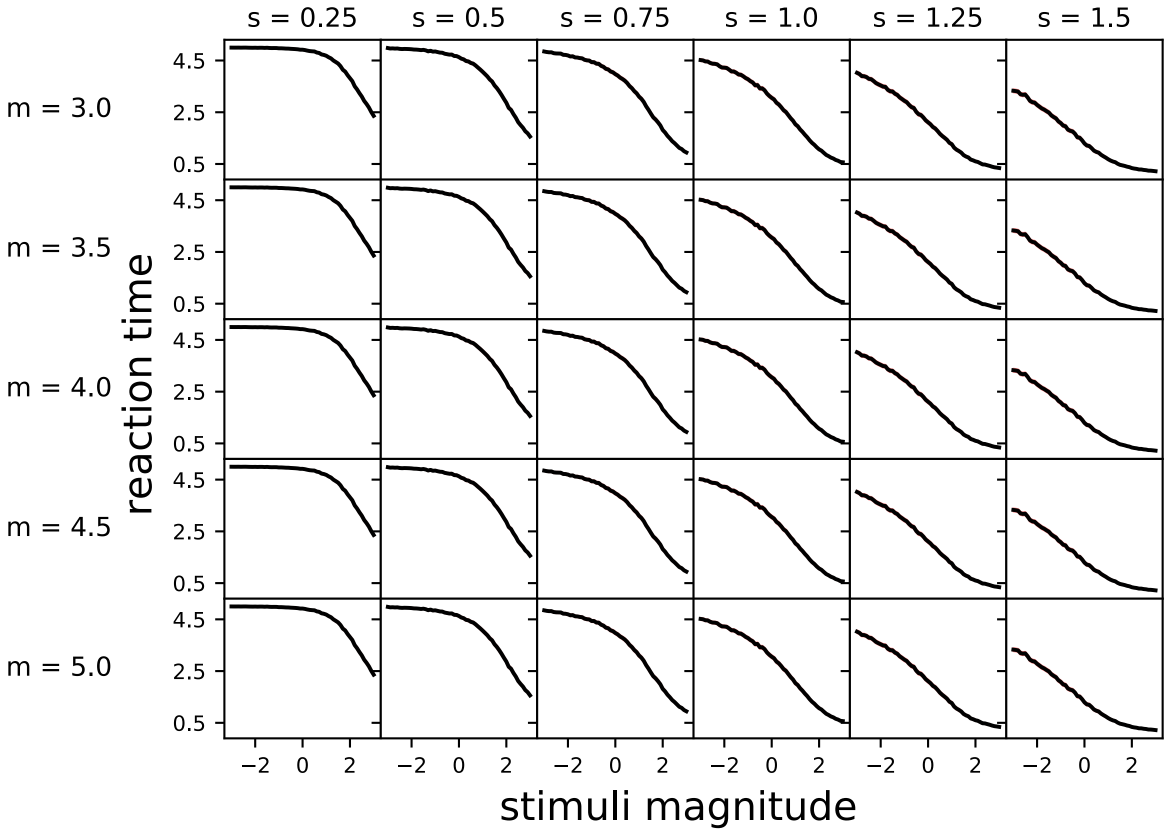

Supplement: S2 Fig — Simulation parameters were: prior mean x¯p,i=1.5 and variance σp,i2=5, observation noise variance σa,i2=2, temporal cost γ = 0.9. Non-decision time was implicitly zero. and simulation timestep dt = 5 × 10^−3^. Lines are the mean reaction time for 104 simulations, 95% confidence intervals are shown as red shading (mostly invisible because smaller than the linewidth). (PDF) [file pcbi.1010523.s005.pdf]
